# Peptides with Diverse Functions from Scorpion Venom: A Great Opportunity for the Treatment of a Wide Variety of Diseases

**DOI:** 10.52547/ibj.3863

**Published:** 2022-11-26

**Authors:** Narges Pashmforoosh, Masoumeh Baradaran

**Keywords:** Antimicrobial peptides, Scorpion venom peptide, Ion channels blocker

## Abstract

Toxicology Research Center, Medical Basic Sciences Research Institute, Ahvaz Jundishapur University of Medical Sciences, Ahvaz, Iran

The venom glands are a rich source of biologically important peptides with pharmaceutical properties. Scorpion venoms have been identified as a reservoir for components that might be considered as great candidates for drug development. Pharmacological properties of the venom compounds have been confirmed in the treatment of different disorders. Ion channel blockers and AMPs are the main groups of scorpion venom components. Despite the existence of several studies about scorpion peptides, there are still valuable components to be discovered. Additionally, owing to the improvement of proteomics and transcriptomics, the number of peptide drugs is steadily increasing, which reflects the importance of these medications. This review evaluates available literatures on some important scorpion venom peptides with pharmaceutical activities. Given that the last three years have been dominated by the COVID-19 from the medical/pharmaceutical perspective, scorpion compounds with the potential against the *coronavirus *2 (SARS-CoV-2) are discussed in this review.

## INTRODUCTION

Biologically active peptides can be found in natural resources, including bacteria, fungi, plants, and animals. These peptides, in comparison with their human counterparts, have been demonstrated to possess therapeutic properties, including higher selectivity, potency, stability in in vivo conditions, all of which are required for therapeutic development^[^^1^^]^. Venom of the venomous animals are a great source of peptides with therapeutic properties, which exhibit variable function and show high selectivity and specificity to human target cells^[^^2^^-^^4^^]^. For more than two decades, scorpion venoms have been studied to identify and characterize the important compounds in the venom gland. Findings have revealed the pharmacological effects of many peptides originated from scorpion venom^[^^5^^]^.

Scorpion venom contains a complex mixture of several low-molecular-weight peptides, mucus, oligopeptides, nucleotides, amino acids, enzymes, lipids, mucoproteins, biogenic amines and other unknown substances^[^^6^^,^^7^^]^. Different peptides, including neurotoxins, cytotoxins, hemotoxins, and AMPs, have also been detected in the venom of scorpions. Neurotoxins have been developed for neurological paralysis of the prey of scorpion and as a defense mechanism against predators^[^^6^^]^. In the venom, these toxins deliver neurotransmitters from the autonomic nervous system, mainly the sympathetic nervous system. Accordingly, some vital functions of the cardiovascular, respiratory, and neuromuscular systems may be quickly influenced by neurotoxins^[^^8^^]^. Sometimes, envenomation leads to cardiac and hemodynamic changes, together with cardiogenic shock and pulmonary edema as a main factor of death^[^^9^^]^.

**Scorpion venom peptides with therapeutic potentials**

Scorpion and its venom have been applied in traditional medicine in China, India, and Africa for thousands of years^[^^10^^]^. Scorpion venom is famous for its deadly effects on cells, tissues, and organisms. However, a significant number of scorpion venom peptides have displayed properties that make them suitable candidates for drug development. Scorpion toxins target ion channels, including sodium, potassium, chloride, and calcium. Therefore, they have important effects on excitable cells^[^^6^^]^ and could be considered for designing drugs in the cardiac diseases^[^^11^^]^, autoimmune diseases^[^^12^^]^, and different types of cancer^[^^13^^]^. Moreover, some antibacterial and antifungal properties have been attributed to different scorpion venom peptides. Generally, these peptides are classified into two groups: DBPs^[^^14^^]^ and NDBPs^[^^15^^]^. DBPs are the main scorpion venom groups containing three to four disulﬁde bridges with neurotoxic effects on scorpion stung victims, since they mostly affect membrane ion channels in the excitable and non-excitable cells^[^^14^^]^. NDBPs compared to DBPs, are small peptides having frequent biological targets^[^^14^^]^. The scorpion toxins, which could bind specifically to mammalian sodium channels, are composed of 61 to 76 amino acid residues in length, and their structures are stabilized by four disulfide bridges^[^^16^^]^. These peptides are grouped into two classes, α- and β-NaTxs^[^^17^^]^. Binding the α-NaTxs to sodium channels results in the slow inactivation of the channels, whereas β-NaTxs interaction with Na^+^ channels causes a shift in the voltage dependence of sodium channel activation to more negative membrane potentials, leading to channel inactivation^[^^18^^]^. Potassium channel toxins, which are characterized by 23 to 64 residues in the length and three or four disulfide bridges, can modulate K^+^ channels^[^^19^^]^. All identified KTx are grouped into α-, β-, γ-, κ-, δ-, λ-^[^^20^^]^, and ε-KTx^[^^21^^]^ subfamilies. The α-KTxs family, the most important family of KTxs, is divided into more than 31 subfamilies^[^^19^^,^^22^^]^. Another family of scorpion toxins are peptides that modulate calcium channels^[^^23^^]^. Kurtoxin^[^^24^^]^ and Kurtoxin-like^[^^25^^]^ peptides are calcium channel toxins that bind to T-type voltage-gated calcium channels. Calcin family of peptides includes maurocalcin, imperacalcin, hadrucalcin, vejocalcin, opicalcin1, opicalcin2, urocalcin, and hemicalcin. These toxins have high affinity to RyR1-3, particularly RyR1, enhancing their activity by inducing subconductance states on the RyR channels^[^^26^^,^^27^^]^. Some peptides extracted from the scorpion venom glands are Cl^-^ channel blockers^[^^28^^]^. Chlorotoxin is the first known peptides with high affinity to Cl^-^ channels^[^^29^^]^ in human astrocytoma and glioma cells^[^^30^^,^^31^^]^, specially ClC-3 channel^[^^32^^]^. GaTx1 and GaTx2 originating from the venom of *Leiurus quinquestriatus* scorpion have been identified to inhibit the cystic fibrosis transmembrane conductance regulator^[^^33^^]^ and CIC-2 chloride channel^[^^34^^]^, respectively. Below, venom components with pharmacological properties have been described and categorized.

**Antimicrobial peptides**

AMPs are produced by bacteria and mammals^[^^35^^]^. In recent years, numerous AMPs have been identified from a wide variety of animals and plants, as well as bacteria and fungi^[^^36^^]^. Scorpions are found as a great source of AMPs. It has been suggested that antibacterial peptides in scorpion venom can preserve the venom gland from infections or enhance the toxin activity. Numerous AMPs have been reported from scorpion venoms^[^^37^^,^^38^^]^. A large number of NDBPs originated from the scorpion are AMPs with antibacterial, antifungal, antimalarial or antiviral properties^[^^14^^]^. Moreover, comprehensive reviews on various groups of AMPs found in the venom of scorpion species have been studied ^[^^21^^,^^38^^-^^42^^]^. Scorpion AMPs are amphipathic and positively charged peptides and divided into three basic categories: (1) cysteine- containing peptides with disulfide bridges, (2) amphipathic α-helical peptides without cysteine residues, and (3) glycine- or proline-rich peptides^[^^40^^]^. Some important AMPs obtained from scorpions are described below. Scorpion venom peptides with cysteine residues mostly have three or four disulfide bridges and could be categorized according to their interaction with ion (Na^+^, Ca2^+^, K^+^, and Cl^-^) channels^[^^40^^]^ (Table 1). Scorpine is the first cysteine-stabilized α/β fold peptide identified in the *Pandinus imperator* venom and has shown great antibacterial and effective antimalarial activity. It possesses a unique amino acid sequence with the C‐terminal region similar to the insect defensins containing three disulfide bridges and N‐terminal sections like cecropins^[^^43^^]^. Studies have reported two scorpine-like peptides Including opiscorpine (opiscorpine 1-4) from *Opistophtalmus* *carinatus*^[^^44^^]^ and Heteroscorpine-1 from *Heterometrus laoticus*, which all were grouped as long-chain K-channel toxins into one family^[^^45^^]^. Four potent AMPs have already been characterized from *Scorpio* *maurus palmatus*, among which Smp76 was scorpion-like peptide^[^^46^^]^ with antiviral activity^[^^47^^]^. The anti-HCV and anti-dengue virus activities of Smp76 could develop a new potential therapeutic against these flaviviruses^[^^47^^]^. In this regard, the recombinant Smp76 has shown the ability to control the viral infection in cell lines and primary mouse macrophages. Inactivation of the viruses occurs by upregulating the IFN-β expression through initiating interferon regulatory transcription factor 3 phosphorylation, leading to the enhancement of the type-I IFN response and inhibition of viral infection^[^^48^^]^. G-TI, bactridines 1-6, and Cm38 peptide isolated respectively from the venom gland of *Androctonus australis* hector^[^^49^^]^, *Tityus discrepans*^[^^50^^]^, and *Centruroides margaritatus*^[^^51^^]^ have demonstrated high antibacterial activity against a wide range of Gram-positive and Gram-negative bacteria. These peptides have shown dual function as a sodium channel blocker and AMPs. Likewise, Ts1 from TsV venom has been reported as a sodium channel toxin that could inhibit the growth of a variety of fungi^[^^52^^]^. The effect of venom obtained from scorpion TsV was estimated by exposing the murine macrophage cell line to TsV and its toxins (Ts1, Ts2, and Ts6) with or without lipopolysaccharide stimulation. While crude venom (TsV) and other toxins did not produce cytotoxic effects, TsV, Ts1, and Ts6 trigged the release of nitric oxide, IL-6, and TNF-α in J774.1 cells. It has been suggested that Ts2 can promote anti-inflammatory activity through secreting the IL-10^[^^53^^]^. In fact, TsV has an immunomodulatory effect on human T-lymphocyte functions^[^^54^^]^. Apparently, treatment with TsV venom can regulate immune mechanism and also activate MAPKs to combat *Trypanosoma cruzi* infection. It has been recommended that Ts7 fraction from crude TsV contains components that are responsible for generating pro-inflammatory immune responses of *T. cruzi*-infected macrophages^[^^55^^]^. It has also been confirmed that TsV components can induce the production of inflammatory mediators by immune cells against *Toxoplasma gondii *activity^[^^56^^]^. *Mesobuthus eupeus* has shown considerable antimicrobial and antiviral activity. MeuTXKbeta1 (from *M. Eupeus*) is a TsTXKβ-related peptide having unique two-domain peptides, which provides neurotoxic and cytolytic activities for this peptide. Synthetic rMeuTXKbeta1 derived from MeuTXKbeta1 has shown anti-plasmodium activity with minimal toxic effects on bacterium and mouse erythrocytes and poor interaction with K^+^ channels^[^^57^^]^. BmKDfsin3 derived from *M.* *martensii *could inhibit HCV replication through suppressing p38 MAPK signal pathway^[^^58^^]^, and BmKDfsin4 successfully controlled HBV replication^[^^59^^]^ and Gram-positive bacteria in vitro^[^^60^^]^. Three AMPs, including meuPep34, meuAP-18-1, and meuVAP-6, were identified and characterized from the transcriptome of the *M. eupeus *venom gland. The meuAP-18-1 and meuVAP-6 are NDBPs, and meuPep34 is a cysteine-rich defensin-like peptide. Due to similarity in DNA sequence of meuPep34 with other AMPs, it has been suggested that meuPep34 is the most potent antibacterial peptide that could control bacterial biofilm^[^^61^^]^. The majority of NDBPs have amphipathic α-helical structures without cysteine residue, which displays a flexible structure^[^^21^^]^. 

**Table 1:** Scorpion-derived cysteine-rich AMPs

Peptide name	Scorpion species	Target	Biological activity	Ref.
**Scorpine**	*P. imperator*	bacteria (*B. subtilis*,* K. pneumoniae*) and *P. berghei*	Antibacterial, Anti-malarial	^[^^43^^]^
**Opiscorpines 1-4**	*O. carinatus*	Yeasts and bacteria	Antibacterial	^[^^44^^]^
**Heteroscorpine-1**	*H. laoticus*	Bacterial species (no Gram-specificity)	Antibacterial	^[^^45^^]^
**Smp76**	*S. m. palmatus*	Flaviviruses	Antiviral	^[^^46^^]^
**G-TI**	*A. australis*	Gram-positive and -negative bacteria	Antibacterial	^[^^49^^]^
**Bactridines 1-6**	*T. discrepans*	Gram-positive and -negative bacteria	Antibacterial	^[^^50^^]^
**Cm38**	*C. margaritatus*	Gram-negative bacteria	Antibacterial	^[^^51^^]^
**Ts1**	*T. serrulatus*	Filamentous fungi	Antifungal	^[^^52^^]^
**Ts7**	*T. serrulatus*	*T. cruzi*	Antiparasitic	^[^^55^^]^
**Su6-B**	*T. serrulatus*	*T. gondii*	Antiparasitic	^[^^56^^]^
**rMeuTXKβ1**	*M. eupeus*	*Plasmodium*	Antimalarial	^[^^57^^]^
**BmKDfsin3**	*M. martensii*	HCV	Antiviral	^[^^58^^]^
**BmKDfsin4**	*M. martensii*	HBV	Antiviral	^[^^59^^]^
**rBmKDfsin4**	*M. martensii*	Gram-positive bacteria	Antibacterial	^[^^60^^]^
**MeuPep34, MeuVAP-6, MeuAP-18-1**	*M. eupeus*	Gram-positive bacteria	Antibacterial	^[^^61^^]^

To date, various NDBPs shave been isolated from varying scorpion species (Table 2). Meucin-18, meucin-13^[^^62^^]^, and meucin-49^[^^63^^]^ peptides isolated from *M. eupeus* have demonstrated extremely powerful antibacterial activity against a wide range of bacteria, including Gram-positive and Gram-negative bacteria. Meucin-24 and meucin-25^[^^64^^]^ from scorpion *M. eupeus* have capability of preventing the Plasmodium development with no toxicity to mammalian cells. The exclusive activity of these peptides against plasmodium makes them effective candidates for optimizing new antimalarial drug design^[^^64^^]^. MesoLys- C (a c-type lysozyme)^[^^65^^]^ and Me-CLAP (caerin-like AMP)^[^^66^^]^ have been isolated from scorpion *M. eupeus* and are known as potent AMPs. Marcin-18 peptide from *M. martensii* has revealed identical sequence and high homology with AMPs meucin-18, and megicin-18 has indicated significant inhibitory activity against Gram-positive bacteria, particularly clinical antibiotic-resistant strains^[^^67^^]^. BmKbpp originated from *M. martensii* Karsch venom gland can inhibit the antimicrobial activity of both Gram-positive and Gram-negative bacteria with the MIC value ranging from 2.3 μM to 68.2 μM. BmKbpp is also capable of blocking some antibiotics-resistant pathogens growth with low hemolytic activity. It has been considered as a potent therapeutic candidate for developing new antibiotics^[^^68^^]^. Stigmurin has been discovered from *T. stigmurus* with antifungal and antibacterial activity^[^^69^^]^.

**Table 2:** Scorpion-derived AMPs without cysteine residues

**Peptide name**	**Scorpion species**	**Target**	**Biological activity**	**Ref.**
Meucin-18 and -13	*M. eupeus*	Gram-positive and -negative bacteria	Antibacterial	^[^^62^^]^
Meucin-49	*M. eupeus*	Gram-positive and -negative bacteria	Antibacterial	^[^^63^^]^
Meucin-24 and -25	*M. eupeus*	*Plasmodium*	Antimalarial	^[^^64^^]^
MesoLys-C	Iranian *M. eupeus*	Gram-positive and -negative bacteria	Antibacterial	^[^^65^^]^
Me-CLAP	Iranian *M. eupeus*	Gram-positive and -negative bacteria	Antibacterial	^[^^66^^]^
Marcin-18	*M. martensii*	Gram-positive bacteria	Antibacterial	^[^^67^^]^
BmKbpp	*M. martensii*	Gram-positive and -negative bacteria	Antibacterial	^[^^68^^]^
Stigmurin	*T. stigmurus*	Gram-positive bacteria and fungi	Antibacterial and antifungal	^[^^69^^]^
TsAP-1 and -2	*T. serrulatus*	Gram-positive bacteria and yeast	Antimicrobial	^[^^70^^]^
ToAP2	*T. obscurus*	*C. albicans*, *C. neoformans*, and *M. massiliense*	Antifungal and anti-mycobacterial	^[^^71^^-^^73^^]^
NDBP-5.7 and -5.5	*O. cayaporum*	*Candida spp. *and *M. massiliense*	Antifungal and anti-mycobacterial	^[^^71^^,^^73^^,^^74^^]^
Pandinin 1 and 2	*P. imperator*	Gram-positive bacteria	Antimicrobial	^[^^75^^]^
Opistoporins 1 and 2	*O. carinatus*	Gram-negative bacteria and fungi	Antibacterial and antifungal	^[^^76^^]^
Imcroporin	*I. maculates*	Gram-positive bacteria	Antimicrobial	^[^^80^^]^
Vejovine	*V. mexicanus*	Gram-negative bacteria	Antibacterial	^[^^37^^]^
Mucroporin	*L. mucronatus*	Gram-positive bacteria	Antibacterial	^[^^81^^]^
Hp1090	*H. petersii*	HCV	Antiviral	^[^^82^^]^
Hp1036 and Hp1239	*H. petersii*	HSV-1	Antiviral	^[^^83^^]^
Hp1404	*H. petersii*	Gram-positive bacteria	Antibacterial	^[^^84^^]^
BmKn-2	*B. martensii *Kasch	Gram-positive and -negative bacteria	Antibacterial	^[^^88^^]^
Kn2-7	*B. martensii *Kasch	HIV/AIDS	Antiviral	^[^^90^^]^
AamAP1 and 2	*A. amoeruxi*	Gram-positive and -negative bacteria	Antibacterial	^[^^91^^]^
IsCT and 2	*O. madagascariensis*	Gram-positive and -negative bacteria	Antibacterial	^[^^77^^,^^78^^]^
Parabutoporin	Parabuthus schlechteri	Gram-negative bacteria and fungi	Antibacterial and antifungal	^[^^79^^]^

Two linear peptides TsAP-1 and TsAP-2 and their analogues (TsAP-S1 and TsAP-S2) from TsV are recognized as AMPs against Gram-positive bacteria and yeasts^[^^70^^]^. Higher helical content of TsAP-2 (76.47%) and hydrophobic moment (0.51 µH) could empower the TsAP-2 antimicrobial property compared to TsAP-1 (58.82% helix; hydrophobic moment of 0.43 µH)^[^^70^^]^. Besides, antitumoral potential of stigmurin has been reported to be similar to TsAP-2^[^^69^^,^^70^^]^. ToAP2 from *Tityus obscurus* and NDBP-5.7 from *Opisthacanthus cayaporum* were synthesized from cDNA library of these scorpions with antifungal activities against different *Candida spp.* and *Cryptococcus neoformans*. Both peptides can improve cell permeability and modify the morphology of* C. albicans* cells; however, ToAP2 was more efficient in inhibiting filamentation. ToAP2 has been reported as the most effective antimicrobial agent and shown synergic effect with both fluconazole and amphotericin B, while NDBP-5.7 presented a synergic effect with fluconazole only^[^^71^^]^. Previously, it has been reported that ToAP2 peptide has activity against both *Cryptococcus neoformans* and *Candida spp*., as well as restrain *C. albicans* biofilm formation^[^^72^^]^. Similarly, ToAP2 and NDBP-5.5 can prevent *Mycobacterium massiliense* strains as an antimycobacterial agent in vitro and in vivo^[^^73^^,^^74^^]^. 

Cationic venom peptides, i.e. Pandinin 1 and 2^[^^75^^]^, opistoporins 1 and 2^[^^76^^]^, IsCT^[^^77^^]^, IsCT2^[^^78^^]^, and parabutoporin^[^^79^^]^, from African scorpion have shown antibacterial, antifungal and hemolytic activities. It has been reported that opistoporin 1 and parabutoporin have strong inhibitory potential against the growth of Gram-negative bacteria^[^^76^^]^, while the peptides pandinins 1 and 2, IsCT, and IsCT2 have high antimicrobial effect on Gram-positive bacteria^[^^75^^,^^77^^,^^78^^]^. Imcroporin extracted from *Isometrus maculates* venom has shown potency against Gram-positive bacteria and mainly against antibiotic-resistant bacteria, including methicillin-resistant *Staphylococcus aureus; *thus, it could be considered as a potent candidate for developing new and effective antibiotic drugs^[^^80^^]^. Vejovine from *Vaejovis mexicanus* has low cytotoxicity on human erythrocytes and affects the growth of Gram-negative bacteria, as well as can be used against multidrug-resistant bacteria^[^^37^^]^. Mucroporin is a cationic peptide isolated from *Lychas mucronatus* showed inhibitory effect against Gram-positive bacteria^[^^81^^]^. 

Three α-helical peptides (Hp1090, Hp1036, and Hp1239) derived from *Heterometrus petersii* have shown antiviral activity. Hp1090 directly inhibits HCV infection with an IC_50_ of 7.62 μg/ml (5.0 μM) in vitro^[^^82^^]^. Both Hp1036 and Hp1239 effectively inhibit the activity of HSV-1 through preventing the viral attachment and entry stages^[^^83^^]^. The cationic AMP Hp1404 extracted from the scorpion *H. petersii* venom revealed a specific inhibitory activity against Gram-positive bacteria such as *Staphylococcus* *aureus*^[^^84^^]^. Interestingly, *S. aureus* has not shown resistance to this peptide. Hp1404 has a low toxicity in both mammalian cells and mice^[^^84^^]^. Antimicrobial assays have also verified that Hp1404 was more potent against carbapenem-resistant *Acinetobacter baumannii*, than other examined peptides^[^^85^^]^. Peptides isolated from Hp1404 have indicated antimicrobial activity against Gram-positive and Gram-negative bacteria, particularly multidrug-resistant *A. baumannii*^[^^86^^]^. In comparison to Hp1404 have shown less toxicity and higher antibiofilm effect^[^^86^^]^. Several analogues of Hp1404 were synthesized and theie antimicrobial activity was assessed. Hp1404-T1e was selected as the most potent antibacterial and antibiofilm analogue, especially against multidrug-resistant *Pseudomonas aeruginosa* strains^[^^87^^]^. 

BmKn-2 peptide from *Buthus martensii* Karsch is characterized as a strong AMP against both Gram-positive and Gram-negative bacteria^[^^88^^]^. BmKn-2 peptide has also demonstrated antitumoral activity against human oral cancer cells^[^^89^^]^. Kn2-7 peptide was originated from BmKn-2 by exchanging serine for glycine and alanine for arginine or lysine to improve its AMP activity. Kn2-7 could potentially prevent HIV-1 infection by direct interaction with HIV-1 envelope. This peptide has shown low cytotoxicity to host cells by a selective index of 13.93^[^^90^^]^. Apart from Kn2-7, both mocroporin-M1 and BmKn2 have been recognized as potent anti-HIV-1 peptides^[^^90^^]^. AamAP1 and AamAP2 extracted from *Androctonus amoeruxi* has shown inhibitory activity against Gram-positive and -negative bacteria^[91]^. Recently, this peptide has been applied as a platform for synthesizing a novel peptide named AamAP1-Lysine with improved antibacterial activity and reduced cytolytic activity^[^^92^^]^. Further evaluation reflected that both AamAP1-Lysine^[^^93^^]^ and A3^[^^94^^]^, another analogue of AamAP1, when administrated with several common antibiotics can enhance antimicrobial effects of those antibiotics against multidrug-resistant strains of bacteria.

Some scorpion peptides are cysteine-rich (Table 3). Serrulin peptide identified in the hemolymph of the TsV scorpion has a high percentage of glycine residues (G), similar to the glycine-rich peptides from spiders^[^^95^^]^. Serrulin has shown a wide range of antimicrobial activity with no hemolytic activity against human erythrocytes^[^^95^^]^. Parabutoporin from *Parabuthus schlechteri* venom has been characterized as a 45-mer lysine-rich peptide that can be active against a wide spectrum of bacteria and fungi^[^^79^^]^. In some cases, adding special amino acid fragments to a peptide can improve its antimicrobial activity. For instance, Ctry2459 established from the cDNA peptide library of *Chaerilus tryznai* could inhibit HCV initial infection, but due to inadequate peptide activity, the viral infection could not be suppressed properly. However, designed histidine-rich peptides Ctry2459-H2 and Ctry2459-H3 could inhibit the viral activity. In addition, both peptides display lower pharmacological toxicities than the wild-type peptide^[^^96^^]^. Eval418 from *Euscorpiops validus* venom could control the initiation of HSV-1 infection, but modified Eval418-FH5 peptide with histidine-rich improved antiviral activity^[^^97^^]^. According to the World Health Organization estimation, antibiotic resistance is a major threat to global public health, which could cause about 10 million deaths each year by 2050^[^^98^^]^. Therefore, discovering alternative antibiotics from natural AMPs might be the most promising action for treating multidrug-resistant pathogen infections^[^^99^^]^.

**Table 3:** Scorpion-derived amino acid-rich AMPs

Peptide name	Scorpion species	Target	Biological activity	Amino acid	Ref.
**Serrulin**	*T. serrulatus*	Gram-positive and -negative bacteria, fungi, yeast	Antibacterial/ antifungal	Glycine	^[^^92^^]^
					
**Parabutoporin (PP)**	*P. schlechteri*	Gram-positive and -negative bacteria, fungi	Antibacterial, antifungal	Lysin	^[^^91^^]^
					
**Ctry2459-H2, Ctry2459-H3**	*C. tryznai*	HCV	Antiviral	Histidine	^[^^93^^]^
					
**Eval418-FH5**	*E. validus*	HSV-1	Antiviral	Histidine	^[^^94^^]^

**Anti-SARS-CoV peptides**

Following the COVID-19 epidemic, research was conducted on the components of the scorpion venom gland as the antiviral compounds for the treatment of COVID-19. Due to antiviral effects of some scorpion venom peptides on some members of Coronaviridae family, these peptides were selected to further evaluate their effects on SARS-CoV-2 virus^[^^100^^]^. Some investigations have also been conducted to assess the potential of other antiviral compounds derived from the venom gland of scorpions^[^^101^^]^. There are several AVPs with highly promising anti-SARS-CoV-2 activities identified from scorpion venom. In an analysis in bioinformatics manner, six mutants of meucin-18, a scorpion venom peptide with strong antimicrobial effect, was designed through the following mutations: A9T, H4Y, A9S, H4F, K7H, and (A9T + H4F). All mutated peptides originated from meucin-18 were docked to the RBD domain of the spike protein of SARS-CoV-2 virus to investigate the interactions with the RBD domain, an important domain of SARS-CoV-2 spike protein. COVID-19 disease begins to develop as a result of interaction between RBD domain and the ACE2 receptor in human cells. A9T mutant (or FFGHLFKLTTKIIP SLFQ) of meucin-18 has shown the best reaction to the RBD domain of the spike protein, even better than native meucin-18. Furthermore, this peptide could change the native conformation of the RBD domain of spike protein. Since the successful interaction with the RBD domain can prevent the interaction of ACE2 receptor with this domain and leads to the unsuccessful entry of the virus into the cell, this protein can be considered as a drug for the treatment of COVID-19^[^^102^^]^. A mutant version of mucroporin called mucroporin-M1 (LFRLIKSL IKRLVSAFK) was designed through G3R, P6K, G10K, and G11R mutations. Mucroporin-M1 was more potent antiviral compound than mucroporin^[^^103^^]^. Mucroporin-M1 has demonstrated activity against SARS-CoV virus along with MERS-CoV, HBV, and influenza H5N1 viruses^[^^103^^,^^104^^]^. After binding of mucroporin-M1 to the SARS-CoV virus through a direct virucidal effect, the strong electrostatic affinity of mucroporin-M1 leads to the destruction of the virus envelope and subsequently decreases viral infectivity^[^^105^^]^. Mucroporin-M1 has direct virucidal action with EC50 of 14.46 μg/ml (7.12 μM) against SARS-CoV^[^^106^^]^. Considering the anti-SARS-CoV activity determined for mucroporin-M1, an anti-SARA-CoV potential is also suggested for this peptide^[^^101^^]^. 

**Cancer therapeutics**

The International Agency for Research on Cancer has recently reported a raise in the global cancer burden to 19.3 million new cases and 10.0 million deaths in 2020^[107]^. An increasing number of in vitro and in vivo studies have shown that scorpion venoms and toxins can decrease cancer growth, induce apoptosis and inhibit cancer progression and metastasis^[^^10^^]^. Thus, scorpion venom is used to treat various cancers such as human neuroblastoma, leukemia, glioma, brain tumor, breast cancer, melanoma, prostate cancer, and human lung adeno-carcenomas^[^^108^^]^. Studies have also suggested that scorpion venoms and toxins are applied as alternative treatments in cancer and metastasis therapy^[^^10^^,^^109^^]^. 

**Table 4:** Scorpion-derived anticancer peptides

Peptide name	Scorpion species	Target	Biological activity	Ref.
**CTX**	*L. quinquestriatus*	glioma cells	Anti-invasive, Antimetastasis	^[^^109^^]^
				
**BmKn-2**	*M. martensii*	HSC4 and KB cells	Apoptosis induction	^[^^120^^]^
				
**BmK AGAP**	*B. martensi*	Breast cancer cell	Anti-invasive Migration inhibition	^[^^121^^]^
				
**Bengalin**	*H. bengalensis*	Leukemic cells	Cell death through autophagy Apoptosis induction	^[^^122^^]^
				
**TsAP-1, TsAP-2**	*T. serrulatus*	Human cancer cell lines	Anti-proliferative	^[^^70^^]^
				
**Stigmurin, TsAP-2**	*T. stigmurus*	Human cancer cell lines	Anti-proliferative	^[^^123^^]^
				
**Neopladine 1, Neopladine 2**	*T. discrepans*	Human breast carcinoma	Apoptosis induction	^[^^124^^]^
				
**Margatoxin**	*C. margaritatus*	Human lung adenocarcinoma	Anti-proliferative	^[^^125^^]^
				
**Mauriporin**	*A. mauritanicus*	Prostate cancer cell lines	Anti-proliferative	^[^^113^^]^

Ample evidence has emphasized that ion channel dysfunction is related to cancer development^[^^110^^,^^111^^]^. Scorpion venoms contain peptide toxins that can modify the activity of voltage-gated Na^+^/K^+^ channels. Consequently, these toxins could change the activity of onco-channels^[^^13^^]^, which are vital for pharmaceutical industry in terms of drug design and development^[^^112^^]^ (Table 4). 

CTX, a chlorotoxin and a small neurotoxin of 36 amino acids isolated from the venom of the scorpion *Leiurus quinquestriatus*, is known as the first extracted chloride channel blocker^[^^29^^,^^109^^]^. Further study has revealed that chlorotoxin could exclusively bind to the surface of glioma cells and show an anti-invasive effect due to interacting with MMP-2 isoforms, as the main CTX receptor, on the surface of glioma cells^[^^113^^]^. Since its discovery, CTX has been linked to nanoparticles, radioisotopes, and fluorescent molecules^[^^114^^]^. For instance, “Tumor paint” is a complex of CTX-Cy5.5 (a near-infrared fluorescent dye) to spot cancer foci and metastases noninvasively and under simulated surgical operating^[^^115^^]^. It has been shown that CTX-modified liposomes are capable of increasing the absorption of doxorubicin hydrochloride in breast tumor 4T1 cells *in vitro* through MMP-2. The CTX modification could improve the targeting ability in the metastatic breast cancer *in vivo*^[^^116^^]^. Recent data have suggested that CTX could bind the endocytic receptor NRP1, leading to the enhanced drug uptake and improved antitumor activity *in vivo*^[^^117^^]^. Besides, three new chlorotoxin-like CTX, including meuCl14, meuCl15, and meuCl16, were identified in the transcriptome of *M. eupeus* venom gland with high sequence identity (71.4%) with chlorotoxin. The meuCl14 has been shown to be a promising candidate in cancer drug delivery systems^[^^118^^]^. BmKn-2, the extracted peptide from *M. martensii* Karsch, can induce apoptosis in HSC-4 at IC_50_ of 29 μg/ml by modulating the expression levels of caspase-3, -7, and -9, and BCL-2, as well as exhibit strong antibacterial activity^[^^89^^]^. One study has revealed that BmKn-2 stimulates apoptosis in HSC4 and KB cells through the activation of tumor suppressor p53^[^^119^^]^. The anticancer assessment of *Buthus martensi* (BmK) venom has effectively shown the induction of apoptosis in malignant glioma U251-MG cells. BmK venom induces the cell death specifically in the cultured malignant glioma U251-MG cells at a concentration of 10 mg/ml. After the incubation of U251-MG cells with BmK venom for 32 and 40 h, 36.2% and 63.1% of the cells showed apoptosis, respectively. Furthermore, BmK venom could significantly inhibit the tumor growth in vivo, which was assessed using U251-MG tumor xenografts in the severe combined immunodeficiency mice^[^^120^^]^. The antitumor-analgesic peptide BmK AGAP isolated from *B. martensii* inhibits breast cancer cell stemness, EMT, migration, and invasion by reducing PTX3 through NF-κB and Wnt/β-catenin signaling pathways in vitro and in vivo^[^^121^^]^. The influence of *Rhopalurus junceus* venom on the cell viability and apoptosis of MDA-MB-231 human breast carcinoma cell line has shown that the venom of this scorpion contains peptide(s) or protein(s), which is/are responsible for inducing apoptosis seen in MDA-MB-231 cells by modulating the expression of apoptosis-related genes, including *p53*, *bax*, *noxa*, *puma*, *caspase 3*, *p21*, *BCL-2*, and *BCL-x*l^[^^122^^]^. The venom of *Heterometrus bengalensis* displayed antiproliferative, cytotoxic and apoptogenic properties on human leukemic cell lines U937 and K562^[^^123^^]^. Subsequently, a novel protein, Bengalin (72 kDa), was isolated from the same scorpion venom. This protein is responsible for cell death at IC_50_ values of 3.7 and 4.1μg/ml for U937 and K562 cells, respectively. According to evidence, Bengalin might affect human leukemic cells through the induction of apoptosis, by facilitating mitochondrial death cascade, as well as by inhibiting HSPs, suppressing telomerase activity, and initiating DNA damage^[^^124^^]^. Apart from apoptosis properties, the venom components of *H. bengalensis* stimulate a different cell death pathway in the form of autophagy in human leukemic U937 cells^[^^125^^]^. TsV scorpion venom could control the cervical cancer through the induction of antiproliferative and antiapoptotic effects^[^^126^^]^. Besides the antibacterial potency of TsAP-1 and TsAP-2 isolated from *T. serrulatus*, they can prevent the growth of human cancer cell lines^[^^70^^]^. Both stigmurin and TsAP-2 (identical to TsAP-2 from *T. serrulatus*) from *T. stigmurus* venom have shown an antiproliferative effect on tumor cells, as well as an antibacterial activity. Both peptides were able to lower microbial load and inflammation in experimental sepsis^[^^127^^]^. Neopladine-1 and -2, from *T. discrepans* scorpion venom, induced apoptosis in 6.3% and 4.1% of SKBR3 cells, respectively. Apoptosis mechanism through the activation of neopladinas is induced by Fas signaling involving in FasL and BCL-2 expression^[^^128^^]^. MgTX, isolated from the venom gland of *Centruroides margaritatus*, suppressed human lung tumor through blocking Kv1.3. The inhibitory effect of this peptide extensively improved the expression level of p21 (Waf1/Cip1) and reduced the expression level of Cdk4 and cyclin D3. Injection of MgTX into nude mice reduced the tumor volume^[^^129^^]^. Mauriporin is a novel NDBP from the venom gland of scorpion *Androctonus* *mauritanicus* that can be effective in the control of prostate cancer. It has been found that synthetic mauriporin can inhibit the proliferation of prostate cancer cell lines at IC_50_ 4.4–7.8 l M^[^^130^^]^. Studies on the scorpion *Odontobuthus doriae* have confirmed the ability of its venom to inhibit DNA synthesis in proliferating human breast cancer cells (MCF-7)^[^^131^^]^ and human neuroblastoma cells^[^^132^^]^. 

**Analgesic peptides**

Previous studies have revealed that Nav channel deregulation leads to many human disorders, including chronic neuropathic pain^[^^133^^]^. These voltage-gated Na^+^ channels are possible targets for modification by a variety of scorpion toxin. Accordingly, scorpion toxins may be useful for developing target-specific analgesics^[^^133^^] ^(Table 5). Some studies have shown that sodium channel subtypes Nav1.7, Nav1.8, and Nav1.9 play a significant role in the transmission of nociceptive signals^[^^134^^]^. Some analgesic peptides have been identified in the venom glands of various scorpion species. *Buthus martensii* Karsch is a widely studied species whose venom has several neuroactive peptides, including analgesic peptides^[^^135^^]^. One of the peptides extracted from *B. martensii* Karsch with analgesic property is BmK AS, which has shown analgesic activity in an animal inflammatory pain model by modulating Nav1.3 channel^[^^136^^]^. 

BmK-YA has been found as an enkephalin-like peptide that could regulate mammalian opioid receptors. The most affected subunit is δ-subtype, which displays a pharmacological profile different from morphine activity^[^^137^^]^. Another peptide, BmK AngM1, has an analgesic effect on mice at the dose of 0.8 mg/kg (63% inhibition efficiency), but the LD_50_ was higher than 50 mg/kg^[^^138^^]^. BmK AGAP, the anticancer peptide mentioned above, inhibited inflammation-associated pain and had an MAPK-mediated mechanism involving in pain-associated behavior^[^^139^^]^. AGAP also alters Nav1.7 channel^[^^140^^]^. ANEP has shown the same activity in a mouse-twisting model and the hot plate assay by inhibiting Nav1.7 channel^[^^141^^]^. BmK AGP-SYPU1^[^^142^^]^ and BmK AGP-SYPU2, two analgesic peptides^[^^143^^]^ extracted from a Chinese scorpion, are significantly activated by adding the arginine^[^^144^^]^ and glycine^[^^145^^]^ residues in the C- terminal region, respectively. Recombinant **(**rBmKM9) peptide synthesized from BmKM9 has indicated a strong impact on Nav1.4, Nav1.5, and Nav1.7 channels and has been shown to delay sodium channel inactivation^[^^146^^]^. Evidence has revealed that Ser54 in the BmK9 neurotoxin plays an important role in the antinociceptive activity through hydrogen bond formation with its side-chain hydroxyl group^[^^147^^]^. Amm VIII purified from *Androctonus mauretanicus mauretanicus* venom gland is an α-toxin that affects neuronal Na^+^ channel (Nav 1.2) more than the skeletal muscle Na^+^ channel (Nav 1.4) in rats^[^^148^^]^. TsNTxP, a non-toxic protein from TsV, has an antinociceptive effect through stimulating the voltage-gated sodium channels^[^^149^^]^. Furthermore, this protein might modulate glutamate release from the synaptosomes of mouse spinal cord without any evidence of acute adverse effects. Therefore, TsNTxP may be a potent nontoxic drug for the treatment of neuropathic pain^[^^149^^]^. Hetlaxin from *Heterometrus laoticus* venom exhibited both anti-nociceptive and anti-inflammatory properties. This peptide belongs to the alpha-toxin family and has shown high affinity to Kv1.3 potassium channel^[^^150^^]^. Leptucin isolated from *Hemiscorpius lepturus* scorpion possessed analgesic activity without any sign of hemolysis or cytotoxicity on mice. It could, therefore, be considered a potent candidate for designing new analgesic drugs^[^^151^^]^. 

**Table 5:** Scorpion-derived analgesic peptides

**Peptide name**	**Scorpion species**	**Ion channel target**	**Ref.**
BmK AS	*B. martensii* Karsch	Nav 1.3	^[^^136^^]^
BmK-YA	*B. martensii* Karsch	Mammalian opioid receptors ( μ, δ and κ subtype)	^[^^137^^]^
(BmK) AngM1	*B. martensii* Karsch	Nav, Kv	^[^^138^^]^
BmK AGAP	*B. martensii Karsch*	Nav 1.7	^[^^139^^]^
ANEP	*B. martensii Karsch*	Nav1.7	^[^^141^^]^
BmK AGP-SYPU1	*B. martensii Karsch*	-	^[^^142^^]^
BmK AGP-SYPU2	*B. martensii Karsch*	-	^[^^167^^]^
BmKM9 (rBmKM9)	*B. martensii Karsch*	Nav1.4, Nav1.5, Nav1.7	^[^^146^^]^
Amm VIII	*A. mauretanicus*	Nav 1.2	^[^^148^^]^
TsNTxP	*T. serrulatus*	Nav	^[^^149^^]^
Hetlaxin	*H. laoticus*	Kv1.3	^[^^150^^]^
Leptucin	*H. lepturus*	-	^[^^151^^]^

***Bradykinin-Potentiating peptides***

The kinin system-bradykinin is essential to promote vascular permeability and initiate vasodilatation in some arteries and veins^[^^152^^]^. The discovery of bradykinin^[^^153^^]^ and the bradykinin-potentiating peptides^[^^154^^]^ offered a new understanding of cardiovascular pathophysiology. After development of captopril as the first active-site directed inhibitor of ACE, it has been used worldwide to treat human hypertension^[^^155^^]^. In recent years, studies on the bradykinin potentiating-peptides from scorpion venom have improved. The venom of *T. serrulatus *has demonstrated that the bradykinin potentiating factors could influence blood pressure through the inhibition of ACE activity and bradykinin receptor synthesis^[^^156^^]^. Peptide K12, derived from the venom gland of *B. occitanus*, exhibited an obvious physiological potentiation of Beadykininon both guinea pig ileum and rat uterus. This peptide also firmly increases the depressor effect of bradykinin on rats’ arterial blood pressure. Peptide K12 can inhibit ACE without being proteolyzed by the enzyme^[^^157^^]^. Assessment of the C-terminal region of BmKbpp, an AMP from *M. martensii*, has confirmed the bradykinin-potentiating activity of this peptide. The activity of BmKbpp-C is more powerful than BmKbp^[^^68^^]^. Hypotensin or TistH as another peptide originated from *T. stigmurus* has shown bradykinin-potentiating activity. This peptide potentiates the bradykinin activity without showing any cytotoxicity effects. Besides, it inhibits ACE, independently^[^^158^^]^. TsHpt-I, from the venom gland of TsV and its synthetic analogue peptides, were able to potentiate the hypotensive effect of bradykinin^[^^159^^]^. 

***Immunosuppressive toxins***

The Kv1.3 voltage-gated potassium channel regulates membrane potential and calcium signaling in human effector memory T cells that are key mediators of autoimmune diseases such as multiple sclerosis, type 1 diabetes, and rheumatoid arthritis. Thus, Kv1.3 blockers have been considered for the treatment of autoimmune diseases^[^^160^^]^. OSK1 (α-KTx3.7), isolated from the venom gland scorpion *Orthochirus scrobiculosus*, along with the related analogues, was assessed in mice. OSK1 is a powerful blocker of the three types of Kv channel (Kv1.1, Kv1.2, and Kv1.3) with IC_50_ values of 0.6, 5.4, and 0.014 nM, respectively and blockes Ca^2+^-activated KCa3.1 channel with IC_50_ values of 225 nM^[^^161^^]^. Among the OSK1 analogues, [K16,D20]-OSK1 is the most potent blocker on Kv1.3 channel, with an IC_50_ value of 0.003 nM, which is considered for the management of memory-T-cell-mediated immune responses^[^^161^^]^. Vm24, derived from the venom gland of the *Vaejovis mexicanus smithi*, has shown high affinity to Kv1.3 channels in human lymphocytes. Vm24 blocks K^+^ channel, leading to the prevention of Ca^2+^ signaling in human T lymphocytes and T-cell proliferation *in vitro* as well as repressed delayed-type hypersensitivity responses in rats *in vivo*^[^^12^^]^. These properties is used for treating certain autoimmune disorders such as multiple sclerosis, rheumatoid arthritis, and type 1 diabetes diseases^[^^12^^]^. ADWX-1, the new analogue of scorpion toxin BmKTX^[^^162^^]^, could inhibit Kv1.3 channel, 100-fold higher than the native BmKTX peptide^[^^163^^]^. HsTX1, isolated from the scorpion *Heterometrus spinnifer* and its analogues, PEG-HsTX1 [R14A] and HsTX1 [R14A], could block Kv1.3.^[^^164^^]^. It has been found that the Ts6 and Ts15 toxins extracted from TsV inhibit the Kv1.3 channels. Further research has revealed that Ts15 can block the Kv2.1 channel, and both Ts6 and Ts15 toxins can suppress the delayed-type hyper-sensitivity response by T cells after 24 h in vivo^[^^165^^]^. St20 is a DBP that has been found in the venom of *Scorpiops tibetanus*. It is able to prevent the expression of the CD69 (cell surface marker) and the secretion of IL-2, IFN-γ, and TNF-α in the activated human T cells. The animal experiments have shown that St20 lessens the delayed-type hypersensitivity reactions^[^^166^^]^.

**Conclusion**

Venom glands of different species of scorpions are essential sources of bioactive components. The importance of scorpion venom in the production and development of new drugs for the treatment of incurable diseases or for the production of drugs with greater therapeutic effects is undeniable. This study attempted to summarize the current state scorpion-derived peptides with pharmaceutical activities. Certainly, more advanced research is being performed on scorpion peptides and their pharmaceutical effects, which will shed light on therapeutic applications of these peptides in future. 

## DECLARATIONS

### Acknowledgments

Authors gratefully thank the Ahvaz Jundishapur University of Medical Sciences, Ahvaz, Iran for the financial support of this study (grant no. TRC-9906).

### Ethical statement

Not applicable.

### Data availability

Data supporting this article are included within the article.

### Author contributions

MB: conceptualization, analyzing data, and writing final draft; NP: writing first draft, and collecting data.

### Conflict of interest

None declared.

### Funding/support

There is no funding supported this project.

## References

[1] Muttenthaler M, King GF, Adams DJ, Alewood PF (2021). Trends in peptide drug discovery. Nature reviews drug discovery..

[2] Ma R, Mahadevappa R, Kwok HF (2017). Venom-based peptide therapy: insights into anti-cancer mechanism. Oncotarget.

[3] Lim HN, Baek SB, Jung HJ (2019). Bee venom and its peptide component melittin suppress growth and migration of melanoma cells via inhibition of PI3K/AKT/mTOR and MAPK pathways. Molecules (Basel, Switzerland).

[4] Ejaz S, Hashmi FB, Malik WN, Ashraf M, Nasim FU, Iqbal M (2018). Applications of venom proteins as potential anticancer agents. Protein and peptide letters.

[5] Suhas R (2022). Structure, function and mechanistic aspects of scorpion venom peptides-A boon for the development of novel therapeutics. European journal of medicinal chemistry reports.

[6] Bosmans F, Tytgat J (2007). Voltage-gated sodium channel modulation by scorpion α-toxins. Toxicon.

[7] Gwee MC, Nirthanan S, Khoo HE, Gopalakrishnakone P, Kini RM, Cheah LS (2002). Autonomic effects of some scorpion venoms and toxins. Clinical and experimental pharmacology and physiology.

[8] Chippaux JP (2012). Emerging options for the management of scorpion stings. Drug design, development and therapy.

[9] Cupo P (2015). Clinical update on scorpion envenoming. Revista da sociedade brasileira de medicina tropical.

[10] Ding J, Chua PJ, Bay BH, Gopalakrishnakone P (2014). Scorpion venoms as a potential source of novel cancer therapeutic compounds. Experimental biology and medicine.

[11] Hodgson WC, Isbister GK (2009). The application of toxins and venoms to cardiovascular drug discovery. Current opinion in pharmacology.

[12] Varga Z, Gurrola-Briones G, Papp F, de la Vega RCR, Pedraza-Alva G, Tajhya RB (2012). Vm24 a natural immunosuppressive peptide, potently and selectively blocks Kv1 3 potassium channels of human T cells. Molecular pharmacology.

[13] Díaz-García A, Varela D (2020). Voltage-Gated K+/Na+ channels and scorpion venom toxins in cancer. Frontiers in pharmacology.

[14] Almaaytah A, Albalas Q (2014). Scorpion venom peptides with no disulfide bridges: a review. Peptides.

[15] Zeng XC, Corzo G, Hahin R (2005). Scorpion venom peptides without disulfide bridges. IUBMB life.

[16] Possani LD, Becerril B, Delepierre M, Tytgat J (1999). Scorpion toxins specific for Na+‐channels. European journal of biochemistry.

[17] Rodríguez de la Vega RC, Possani LD (2005). Overview of scorpion toxins specific for Na+ channels and related peptides: biodiversity, structure–function relationships and evolution. Toxicon.

[18] Catterall WA (1992). Cellular and molecular biology of voltage-gated sodium channels. Physiological reviews.

[19] Rodríguez de la Vega RC, Possani LD (2004). Current views on scorpion toxins specific for K+-channels. Toxicon.

[20] Cremonez CM, Maiti M, Peigneur S, Cassoli JS, Dutra AA, Waelkens E, Lescrinier E, Herdewijn P, de Lima ME, Pimenta AM, Arantes EC, Tytgat J (2016). Structural and functional elucidation of peptide Ts11 shows evidence of a novel subfamily of scorpion venom toxins. Toxins (Basel).

[21] Ahmadi S, Knerr JM, Argemi L, Bordon KC, Pucca MB, Cerni FA, Arantes EC, Çalışkan F, Laustsen AH (2020). Scorpion venom: detriments and benefits. Biomedicines.

[22] Soorki MN, Galehdari H, Baradaran M, Jalali A (2016). First venom gland transcriptomic analysis of Iranian yellow scorpion “Odonthubuthus doriae” with some new findings. Toxicon.

[23] Norton RS, McDonough SI (2008). Peptides targeting voltage-gated calcium channels. Current pharmaceutical design.

[24] Chuang RSI, Jaffe H, Cribbs L, Perez-Reyes E, Swartz KJ (1998). Inhibition of T-type voltage-gated calcium channels by a new scorpion toxin. Nature neuroscience.

[25] Olamendi-Portugal T, García BI, López-González I, Van Der Walt J, Dyason K, Ulens C (2002). Two new scorpion toxins that target voltage-gated Ca2+ and Na+ channels. Biochemical and biophysical research communications.

[26] Lanner JT, Georgiou DK, Joshi AD, Hamilton SL (2010). Ryanodine receptors: structure, expression, molecular details, and function in calcium release. Cold spring harbor perspectives in biology.

[27] Xiao L, Gurrola GB, Zhang J, Valdivia CR, SanMartin M, Zamudio FZ, Zhang L, Possani LD, Valdivia HH (2016). Structure-function relationships of peptides forming the calcin family of ryanodine receptor ligands. Journal of general physiology.

[28] Housley DM, Housley GD, Liddell MJ, Jennings EA (2017). Scorpion toxin peptide action at the ion channel subunit level. Neuropharmacology.

[29] DeBin JA, Maggio JE, Strichartz GR (1993). Purification and characterization of chlorotoxin, a chloride channel ligand from the venom of the scorpion. American journal of physiology.

[30] Olsen ML, Schade S, Lyons SA, Amaral MD, Sontheimer H (2003). Expression of voltage-gated chloride channels in human glioma cells. The journal of neuroscience : the official journal of the society for neuroscience.

[31] Ullrich N, Gillespie GY, Sontheimer H (1996). Human astrocytoma cells express a unique chloride current. Neuroreport.

[32] Qin C, He B, Dai W, Lin Z, Zhang H, Wang X, Wang J, Zhang X, Wang G, Yin L, Zhang Q (2014). The impact of a chlorotoxin-modified liposome system on receptor MMP-2 and the receptor-associated protein ClC-3. Biomaterials.

[33] Fuller MD, Thompson CH, Zhang ZR, Freeman CS, Schay E, Szakács G, Bakos E, Sarkadi B, McMaster D, French RJ, Pohl J, Kubanek J, McCarty NA (2007). State-dependent inhibition of cystic fibrosis transmembrane conductance regulator chloride channels by a novel peptide toxin. Journal of biological chemistry.

[34] Thompson CH, Olivetti PR, Fuller MD, Freeman CS, McMaster D, French RJ, Pohl J, Kubanek J, McCarty NA (2009). Isolation and characterization of a high affinity peptide inhibitor of ClC-2 chloride channels. Journal of biological chemistry.

[35] Maróti G, Kereszt A, Kondorosi E, Mergaert P (2011). Natural roles of antimicrobial peptides in microbes, plants and animals. Research in microbiology.

[36] Reddy KV, Yedery RD, Aranha C (2004). Antimicrobial peptides: premises and promises. International journal of antimicrobial agents.

[37] Hernández-Aponte CA, Silva-Sanchez J, Quintero-Hernández V, Rodríguez-Romero A, Balderas C, Possani LD, Gurrola GB (2011). Vejovine, a new antibiotic from the scorpion venom of Vaejovis mexicanus. Toxicon.

[38] Ortiz E, Gurrola GB, Schwartz EF, Possani LD (2015). Scorpion venom components as potential candidates for drug development. Toxicon.

[39] Ghosh A, Roy R, Nandi M, Mukhopadhyay A (2019). Scorpion venom–toxins that aid in drug development: a review. International journal of peptide research and therapeutics.

[40] Harrison PL, Abdel-Rahman MA, Miller K, Strong PN (2014). Antimicrobial peptides from scorpion venoms. Toxicon.

[41] Hmed B, Serria HT, Mounir ZK (2013). Scorpion peptides: potential use for new drug development. Journal of toxicology.

[42] Wang X, Wang G (2016). Insights into antimicrobial peptides from spiders and scorpions. Protein and peptide letters.

[43] Conde R, Zamudio FZ, Rodríguez MH, Possani LD (2000). Scorpine, an anti-malaria and anti-bacterial agent purified from scorpion venom. FEBS letters.

[44] Zhu S, Tytgat J (2004). The scorpine family of defensins: gene structure, alternative polyadenylation and fold recognition. Cellular and molecular life sciences.

[45] Uawonggul N, Thammasirirak S, Chaveerach A, Arkaravichien T, Bunyatratchata W, Ruangjirachuporn W, Jearranaiprepame P, Nakamura T, Matsuda M, Kobayashi M, Hattori S, Daduang S (2007). Purification and characterization of Heteroscorpine-1 (HS-1) toxin from Heterometrus laoticus scorpion venom. Toxicon.

[46] Abdel-Rahman MA, Quintero-Hernandez V, Possani LD (2013). Venom proteomic and venomous glands transcriptomic analysis of the Egyptian scorpion Scorpio maurus palmatus (Arachnida: Scorpionidae). Toxicon.

[47] El-Bitar AMH, Sarhan M, Abdel-Rahman MA, Quintero-Hernandez V, Aoki-Utsubo C, Moustafa MA, Possani LD, Hotta H (2020). Smp76, a scorpine-like peptide isolated from the venom of the scorpion Scorpio maurus palmatus, with a potent antiviral activity against hepatitis C virus and dengue virus. International journal of peptide research and therapeutics.

[48] Ji Z, Li F, Xia Z, Guo X, Gao M, Sun F, Cheng Y, Wu Y, Li W, Ali SA, Cao Z (2018). The scorpion venom peptide Smp76 inhibits viral infection by regulating type-I interferon response. Virologica sinica.

[49] Zerouti K, Khemili D, Laraba-Djebari F, Hammoudi-Triki D (2019). Nontoxic fraction of scorpion venom reduces bacterial growth and inflammatory response in a mouse model of infection. Toxin reviews..

[50] Díaz P, D'Suze G, Salazar V, Sevcik C, Shannon JD, Sherman NE, Fox JW (2009). Antibacterial activity of six novel peptides from Tityus discrepans scorpion venom A fluorescent probe study of microbial membrane Na+ permeability changes. Toxicon.

[51] Dueñas-Cuellar RA, Kushmerick C, Naves LA, Batista IF, Guerrero-Vargas JA, Pires OR Jr, Fontes W, Castro MS (2015). Cm38: a new antimicrobial peptide active against Klebsiella pneumoniae is homologous to Cn11. Protein peptide letters.

[52] Santussi WM, Bordon KCF, Rodrigues Alves APN, Cologna CT, Said S, Arantes EC (2017). Antifungal activity against filamentous fungi of Ts1, a Multifunctional toxin from tityus serrulatus scorpion venom. Frontiers in microbiology.

[53] Zoccal KF, da Silva Bitencourt C, Secatto A, Sorgi CA, Bordon KdCF, Sampaio SV (2011). Tityus serrulatus venom and toxins Ts1, Ts2 and Ts6 induce macrophage activation and production of immune mediators. Toxicon.

[54] Casella-Martins A, Ayres LR, Burin SM, Morais FR, Pereira JC, Faccioli LH, Sampaio SV, Arantes EC, Castro FA, Pereira-Crott LS (2015). Immunomodulatory activity of Tityus serrulatus scorpion venom on human T lymphocytes. Journal of venomous animals and toxins including tropical diseases.

[55] de Oliveira Pimentel PM, de Assis DRR, Gualdrón-Lopez M, Barroso A, Brant F, Leite PG, de Lima Oliveira BC, Esper L, McKinnie SMK, Vederas JC, do Nascimento Cordeiro M, Dos Reis PVM, Teixeira MM, de Castro Pimenta AM, Borges MH, de Lima ME, Machado FS (2021). Tityus serrulatus scorpion venom as a potential drug source for Chagas' disease: Trypanocidal and immunomodulatory activity. Clinical immunology.

[56] de Assis DRR, Pimentel PMO, Dos Reis PVM, Rabelo RAN, Vitor RWA, Cordeiro MDN, Felicori LF, Olórtegui CDC, Resende JM, Teixeira MM, Borges MH, de Lima ME, Pimenta AMC, Machado FS (2021). Tityus serrulatus (scorpion): from the crude venom to the construction of synthetic peptides and their possible therapeutic application against Toxoplasma gondii infection. Frontiers in cellular and infection microbiology.

[57] Zhu S, Gao B, Aumelas A, del Carmen Rodríguez M, Lanz-Mendoza H, Peigneur S, Diego-Garcia E, Martin-Eauclaire MF, Tytgat J, Possani LD (2010). MeuTXKβ1, a scorpion venom-derived two-domain potassium channel toxin-like peptide with cytolytic activity. Biochimica et biophysica acta (BBA)-proteins and proteomics.

[58] Cheng Y, Sun F, Li S, Gao M, Wang L, Sarhan M, Abdel-Rahman MA, Li W, Kwok HF, Wu Y, Cao Z (2020). Inhibitory activity of a scorpion defensin BmKDfsin3 against Hepatitis C virus. Antibiotics (Basel).

[59] Zeng Z, Zhang Q, Hong W, Xie Y, Liu Y, Li W, Wu Y, Cao Z (2016). A scorpion defensin BmKDfsin4 inhibits Hepatitis B virus replication in vitro. Toxins.

[60] Meng L, Xie Z, Zhang Q, Li Y, Yang F, Chen Z, Li W, Cao Z, Wu Y (2016). Scorpion potassium channel-blocking defensin highlights a functional link with neurotoxin. Journal of biological chemistry.

[61] Baradaran M, Jalali A, Naderi Soorki M, Galehdari H (2017). A novel defensin-like peptide associated with two other new cationic antimicrobial peptides in transcriptome of the iranian scorpion venom. Iranian biomedical journal.

[62] Gao B, Sherman P, Luo L, Bowie J, Zhu S (2009). Structural and functional characterization of two genetically related meucin peptides highlights evolutionary divergence and convergence in antimicrobial peptides. FASEB journal.

[63] Gao B, Dalziel J, Tanzi S, Zhu S (2018). Meucin-49, a multifunctional scorpion venom peptide with bactericidal synergy with neurotoxins. Amino acids.

[64] Gao B, Xu J, Rodriguez Mdel C, Lanz-Mendoza H, Hernández-Rivas R, Du W, Zhu S (2010). Characterization of two linear cationic antimalarial peptides in the scorpion Mesobuthus eupeus. Biochimie.

[65] Baradaran M, Jolodar A, Jalali A, Navidpour S, Kafilzadeh F (2011). Sequence analysis of lysozyme C from the scorpion Mesobuthus eupeus venom glands using semi-nested RT-PCR. Iranian red crescent medical journal.

[66] Baradaran M, Jalali A, Jolodar A, Ghasemian S (2012). New caerin-like antibacterial peptide from the venom gland of the Iranian scorpion Mesobuthus eupeus: cDNA amplification and sequence analysis. African journal of biotechnology.

[67] Liu G, Yang F, Li F, Li Z, Lang Y, Shen B, Wu Y, Li W, Harrison PL, Strong PN, Xie Y, Miller K, Cao Z (2018). Therapeutic potential of a scorpion venom-derived antimicrobial peptide and its homologs against antibiotic-resistant Gram-positive bacteria. Frontiers in microbiology.

[68] Zeng XC, Wang S, Nie Y, Zhang L, Luo X (2012). Characterization of BmKbpp, a multifunctional peptide from the Chinese scorpion Mesobuthus martensii Karsch: gaining insight into a new mechanism for the functional diversification of scorpion venom peptides. Peptides.

[69] de Melo ET, Estrela AB, Santos EC, Machado PR, Farias KJ, Torres TM, Carvalho E, Lima JP, Silva-Júnior AA, Barbosa EG, Fernandes-Pedrosa Mde F (2015). Structural characterization of a novel peptide with antimicrobial activity from the venom gland of the scorpion Tityus stigmurus: Stigmurin. Peptides.

[70] Guo X, Ma C, Du Q, Wei R, Wang L, Zhou M, Chen T, Shaw C (2013). Two peptides, TsAP-1 and TsAP-2, from the venom of the Brazilian yellow scorpion, Tityus serrulatus: evaluation of their antimicrobial and anticancer activities. Biochimie.

[71] do Nascimento Dias J, de Souza Silva C, de Araújo AR, Souza JMT, de Holanda Veloso Júnior PH, Cabral WF, da Glória da Silva M, Eaton P, de Souza de Almeida Leite JR, Nicola AM, Albuquerque P, Silva-Pereira I (2020). Mechanisms of action of antimicrobial peptides ToAP2 and NDBP-5 7 against Candida albicans planktonic and biofilm cells. Scientific reports.

[72] Guilhelmelli F, Vilela N, Smidt KS, de Oliveira MA, da Cunha Morales Álvares A, Rigonatto MC, da Silva Costa PH, Tavares AH, de Freitas SM, Nicola AM, Franco OL, Derengowski LD, Schwartz EF, Mortari MR, Bocca AL, Albuquerque P, Silva-Pereira I (2016). Activity of Scorpion Venom-Derived Antifungal Peptides against Planktonic Cells of Candida spp and Cryptococcus neoformans and Candida albicans Biofilms. Frontiers in microbiology.

[73] Marques-Neto LM, Trentini MM, das Neves RC, Resende DP, Procopio VO, da Costa AC, Kipnis A, Mortari MR, Schwartz EF, Junqueira-Kipnis AP (2018). Antimicrobial and chemotactic activity of scorpion-derived peptide, ToAP2, against mycobacterium massiliensis. Toxins.

[74] Trentini MM, das Neves RC, Santos BP, DaSilva RA, de Souza AC, Mortari MR, Schwartz EF, Kipnis A, Junqueira-Kipnis AP (2017). Non-disulfide-bridge peptide 5 5 from the scorpion Hadrurus gertschi inhibits the growth of Mycobacterium abscessus subsp. massiliense. Frontiers in Microbiology.

[75] Corzo G, Escoubas P, Villegas E, Barnham KJ, He W, Norton RS, Nakajima T (2001). Characterization of unique amphipathic antimicrobial peptides from venom of the scorpion Pandinus imperator. Biochemical journal.

[76] Moerman L, Bosteels S, Noppe W, Willems J, Clynen E, Schoofs L, Thevissen K, Tytgat J, Van Eldere J, Van Der Walt J, Verdonck F (2002). Antibacterial and antifungal properties of alpha-helical, cationic peptides in the venom of scorpions from southern Africa. European journal of biochemistry.

[77] Dai L, Yasuda A, Naoki H, Corzo G, Andriantsiferana M, Nakajima T (2001). IsCT, a novel cytotoxic linear peptide from scorpion Opisthacanthus madagascariensis. Biochemical and biophysical research communications.

[78] Dai L, Corzo G, Naoki H, Andriantsiferana M, Nakajima T (2002). Purification, structure–function analysis, and molecular characterization of novel linear peptides from scorpion Opisthacanthus madagascariensis. Biochemical and biophysical research communications.

[79] Remijsen Q, Verdonck F, Willems J (2010). Parabutoporin, a cationic amphipathic peptide from scorpion venom: much more than an antibiotic. Toxicon.

[80] Zhao Z, Ma Y, Dai C, Zhao R, Li S, Wu Y, Cao Z, Li W (2009). Imcroporin, a new cationic antimicrobial peptide from the venom of the scorpion Isometrus maculates. Antimicrobial agents and chemotherapy.

[81] Dai C, Ma Y, Zhao Z, Zhao R, Wang Q, Wu Y, Cao Z, Li W (2008). Mucroporin, the first cationic host defense peptide from the venom of Lychas mucronatus. Antimicrobial agents and chemotherapy.

[82] Yan R, Zhao Z, He Y, Wu L, Cai D, Hong W, Wu Y, Cao Z, Zheng C, Li W (2011). A new natural α-helical peptide from the venom of the scorpion Heterometrus petersii kills HCV. Peptides.

[83] Hong W, Li T, Song Y, Zhang R, Zeng Z, Han S, Zhang X, Wu Y, Li W, Cao Z (2014). Inhibitory activity and mechanism of two scorpion venom peptides against herpes simplex virus type 1. Antiviral research.

[84] Li Z, Xu X, Meng L, Zhang Q, Cao L, Li W, Wu Y, Cao Z (2014). Hp1404, a new antimicrobial peptide from the scorpion Heterometrus petersii. PloS one.

[85] Luo X, Ye X, Ding L, Zhu W, Zhao Z, Luo D, Liu N, Sun L, Chen Z (2021). Identification of the scorpion venom-derived antimicrobial peptide Hp1404 as a new antimicrobial agent against carbapenem-resistant Acinetobacter baumannii. Microbial pathogenesis.

[86] Hong MJ, Kim MK, Park Y (2021). Comparative antimicrobial activity of Hp404 peptide and its analogs against Acinetobacter baumannii. International journal of molecular sciences.

[87] Kim MK, Kang HK, Ko SJ, Hong MJ, Bang JK, Seo CH, Park Y (2018). Mechanisms driving the antibacterial and antibiofilm properties of Hp1404 and its analogue peptides against multidrug-resistant Pseudomonas aeruginosa. Scientific reports.

[88] Zeng XC, Wang SX, Zhu Y, Zhu SY, Li WX (2004). Identification and functional characterization of novel scorpion venom peptides with no disulfide bridge from Buthus martensii Karsch. Peptides.

[89] Tong-ngam P, Roytrakul S, Sritanaudomchai H (2015). BmKn-2 scorpion venom peptide for killing oral cancer cells by apoptosis. Asian pacific journal of cancer prevention.

[90] Chen Y, Cao L, Zhong M, Zhang Y, Han C, Li Q, Yang J, Zhou D, Shi W, He B, Liu F, Yu J, Sun Y, Cao Y, Li Y, Li W, Guo D, Cao Z, Yan H (2012). Anti-HIV-1 activity of a new scorpion venom peptide derivative Kn2-7. PloS one.

[91] Almaaytah A, Zhou M, Wang L, Chen T, Walker B, Shaw C (2012). Antimicrobial/cytolytic peptides from the venom of the North African scorpion, Androctonus amoreuxi: biochemical and functional characterization of natural peptides and a single site-substituted analog. Peptides.

[92] Almaaytah A, Tarazi S, Abu-Alhaijaa A, Altall Y, Alshar'i N, Bodoor K, Al-Balas Q (2014). Enhanced antimicrobial activity of AamAP1-Lysine, a novel synthetic peptide analog derived from the scorpion venom peptide AamAP1. Pharmaceuticals.

[93] 93Almaaytah A, Abualhaijaa A, Alqudah O (2019). The evaluation of the synergistic antimicrobial and antibiofilm activity of AamAP1-Lysine with conventional antibiotics against representative resistant strains of both Gram-positive and Gram-negative bacteria. Infection and drug resistance.

[94] Almaaytah A, Farajallah A, Abualhaijaa A, Al-Balas Q (2018). A3, a scorpion venom derived peptide analogue with potent antimicrobial and potential antibiofilm activity against clinical isolates of multi-drug resistant gram positive bacteria. Molecules.

[95] de Jesus Oliveira T, Oliveira UC, da Silva Junior PI (2019). Serrulin: a glycine-rich bioactive peptide from the hemolymph of the yellow tityus serrulatus scorpion. Toxins (Basel).

[96] Hong W, Zhang R, Di Z, He Y, Zhao Z, Hu J, Wu Y, Li W, Cao Z (2013). Design of histidine-rich peptides with enhanced bioavailability and inhibitory activity against hepatitis C virus. Biomaterials.

[97] Zeng Z, Zhang R, Hong W, Cheng Y, Wang H, Lang Y, Ji Z, Wu Y, Li W, Xie Y, Cao Z (2018). Histidine-rich modification of a scorpion-derived peptide improves bioavailability and inhibitory activity against HSV-1. Theranostics.

[98] O'Neill J (2016). Tackling drug-resistant infections globally: final report and recommendations.

[99] Hassan M, Kjos M, Nes I, Diep D, Lotfipour F (2012). Natural antimicrobial peptides from bacteria: characteristics and potential applications to fight against antibiotic resistance. Journal of applied microbiology.

[100] Chew MF, Poh KS, Poh CL (2017). Peptides as therapeutic agents for dengue virus. International journal of medical sciences.

[101] Mahendran ASK, Lim YS, Fang CM, Loh HS, Le CF (2020). The potential of antiviral peptides as COVID-19 therapeutics. Frontiers in pharmacology.

[102] Mahnam K, Lotfi M, Shapoorabadi FA (2021). Examining the interactions scorpion venom peptides (HP1090, Meucin-13, and Meucin-18) with the receptor binding domain of the coronavirus spike protein to design a mutated therapeutic peptide. Journal of molecular graphics and modelling.

[103] Li Q, Zhao Z, Zhou D, Chen Y, Hong W, Cao L, Yang J, Zhang Y, Shi W, Cao Z, Wu Y, Yan H, Li W (2011). Virucidal activity of a scorpion venom peptide variant mucroporin-M1 against measles, SARS-CoV and influenza H5N1 viruses. Peptides.

[104] Koganti R, Yadavalli T, Shukla D (2019). Current and emerging therapies for ocular herpes simplex virus type-1 infections. Microorganisms.

[105] Kim SH (2018). Challenge for one health: co-circulation of zoonotic h5n1 and H9N2 avian influenza viruses in Egypt. Viruses.

[106] Agarwal G, Gabrani R (2021). Antiviral peptides: identification and validation. International journal of peptide research and therapeutics.

[107] Ferlay J, Ervik M, Lam F, Colombet M, Mery L, Piñeros M, Znaor A, Soerjomataram I, Bray F ( 2020). Global Cancer Observatory: Cancer Today. International Agency for Research on Cancer.

[108] Mishal R, Tahir HM, Zafar K, Arshad M (2013). Anti-cancerous applications of scorpion venom. International journal of biological and pharmaceutical sciences.

[109] Srairi-Abid N, Othman H, Aissaoui D, BenAissa R (2019). Anti-tumoral effect of scorpion peptides: Emerging new cellular targets and signaling pathways. Cell calcium.

[110] Litan A, Langhans SA (2015). Cancer as a channelopathy: ion channels and pumps in tumor development and progression. Frontiers in cellular neuroscience.

[111] Prevarskaya N, Skryma R, Shuba Y (2018). Ion channels in cancer: are cancer hallmarks oncochannelopathies?. Physiological reviews.

[112] Heinen TE, da Veiga ABG (2011). Arthropod venoms and cancer. Toxicon.

[113] Deshane J, Garner CC, Sontheimer H (2003). Chlorotoxin inhibits glioma cell invasion via matrix metalloproteinase-2. The journal of biological chemistry.

[114] Dardevet L, Rani D, Aziz TA, Bazin I, Sabatier JM, Fadl M, Brambilla E, De Waard M (2015). Chlorotoxin: a helpful natural scorpion peptide to diagnose glioma and fight tumor invasion. Toxins.

[115] Veiseh M, Gabikian P, Bahrami SB, Veiseh O, Zhang M, Hackman RC, Ravanpay AC, Stroud MR, Kusuma Y, Hansen SJ, Kwok D, Munoz NM, Sze RW, Grady WM, Greenberg NM, Ellenbogen RG, Olson JM (2007). Tumor paint: a chlorotoxin:Cy5 5 bioconjugate for intraoperative visualization of cancer foci. Cancer research.

[116] Qin C, He B, Dai W, Zhang H, Wang X, Wang J, Zhang X, Wang G, Yin L, Zhang Q (2014). Inhibition of metastatic tumor growth and metastasis via targeting metastatic breast cancer by chlorotoxin-modified liposomes. Molecular pharmaceutics.

[117] McGonigle S, Majumder U, Kolber-Simonds D, Wu J, Hart A, Noland T, TenDyke K, Custar D, Li D, Du H, Postema MHD, Lai WG, Twine NC, Woodall-Jappe M, Nomoto K (2019). Neuropilin-1 drives tumor-specific uptake of chlorotoxin. Cell communication and signaling.

[118] Baradaran M, Jalali A, Soorki MN, Jokar M, Galehdari H (2019). Three new scorpion chloride channel toxins as potential anti-cancer drugs: Computational prediction of the interactions with hMMP-2 by docking and steered molecular dynamics simulations. Iranian journal of pharmaceutical research.

[119] Satitmanwiwat S, Changsangfa C, Khanuengthong A, Promthep K, Roytrakul S, Arpornsuwan T, Saikhun K, Sritanaudomchai H (2016). The scorpion venom peptide BmKn2 induces apoptosis in cancerous but not in normal human oral cells. Biomedicine and pharmacotherapy.

[120] Wang WX, Ji YH (2005). Scorpion venom induces glioma cell apoptosis in vivo and inhibits glioma tumor growth in vitro. Journal of neuro-oncology.

[121] Kampo S, Ahmmed B, Zhou T, Owusu L, Anabah TW, Doudou NR, Kuugbee ED, Cui Y, Lu Z, Yan Q, Wen QP (2019). Scorpion venom analgesic peptide, BmK AGAP Inhibits stemness, and epithelial-mesenchymal transition by down-regulating PTX3 in breast cancer. Frontiers in oncology.

[122] Díaz-García A, Ruiz-Fuentes JL, Rodríguez-Sánchez H, Fraga Castro JA (2017). Rhopalurus junceus scorpion venom induces apoptosis in the triple negative human breast cancer cell line MDA-MB-231. Journal of venom research.

[123] Das Gupta S, Debnath A, Saha A, Giri B, Tripathi G, Vedasiromoni JR, Gomes A, Gomes A (2007). Indian black scorpion (Heterometrus bengalensis Koch) venom induced antiproliferative and apoptogenic activity against human leukemic cell lines U937 and K562. Leukemia research.

[124] Gupta SD, Gomes A, Debnath A, Saha A, Gomes A (2010). Apoptosis induction in human leukemic cells by a novel protein Bengalin, isolated from Indian black scorpion venom: through mitochondrial pathway and inhibition of heat shock proteins. Chemico-biological interactions.

[125] Das Gupta S, Halder B, Gomes A, Gomes A (2013). Bengalin initiates autophagic cell death through ERK-MAPK pathway following suppression of apoptosis in human leukemic U937 cells. Life sciences.

[126] Bernardes-Oliveira E, Farias KJS, Gomes DL, Araújo JMGd, Silva WDd, Rocha HAO (2019). Tityus serrulatus scorpion venom induces apoptosis in cervical cancer cell lines. Evidence-Based complementary and aternative medicine.

[127] Daniele-Silva A, Machado RJ, Monteiro NK, Estrela AB, Santos EC, Carvalho E, Araújo Júnior RF, Melo-Silveira RF, Rocha HA, Silva-Júnior AA, Fernandes-Pedrosa MF (2016). Stigmurin and TsAP-2 from Tityus stigmurus scorpion venom: assessment of structure and therapeutic potential in experimental sepsis. Toxicon.

[128] D'Suze G, Rosales A, Salazar V, Sevcik C (2010). Apoptogenic peptides from Tityus discrepans scorpion venom acting against the SKBR3 breast cancer cell line. Toxicon.

[129] Jang SH, Choi SY, Ryu PD, Lee SY (2011). Anti-proliferative effect of Kv1 3 blockers in A549 human lung adenocarcinoma in vitro and in vivo. European journal of pharmacology.

[130] Almaaytah A, Tarazi S, Mhaidat N, Al-Balas Q, Mukattash TL (2013). Mauriporin, a novel cationic α-helical peptide with selective cytotoxic activity against prostate cancer cell lines from the venom of the scorpion Androctonus mauritanicus. International journal of peptide research and therapeutics.

[131] Zargan J, Umar S, Sajad M, Naime M, Ali S, Khan HA (2011). Scorpion venom (Odontobuthus doriae) induces apoptosis by depolarization of mitochondria and reduces S-phase population in human breast cancer cells (MCF-7). Toxicology in vitro.

[132] Zargan J, Sajad M, Umar S, Naime M, Ali S, Khan HA (2011). Scorpion (Odontobuthus doriae) venom induces apoptosis and inhibits DNA synthesis in human neuroblastoma cells. Molecular and cellular biochemistry.

[133] Cardoso FC, Lewis RJ (2018). Sodium channels and pain: from toxins to therapies. British journal of pharmacology.

[134] Wood JN, Boorman JP, Okuse K, Baker MD (2004). Voltage-gated sodium channels and pain pathways. Journal of neurobiology.

[135] Li Z, Hu P, Wu W, Wang Y (2019). Peptides with therapeutic potential in the venom of the scorpion Buthus martensii Karsch. Peptides.

[136] Liu ZR, Tao J, Dong BQ, Ding G, Cheng ZJ, He HQ (2012). Pharmacological kinetics of BmK AS, a sodium channel site 4-specific modulator on Nav1 3. Neuroscience bulletin.

[137] Zhang Y, Xu J, Wang Z, Zhang X, Liang X, Civelli O (2012). Bm K-YA, an enkephalin-like peptide in scorpion venom. PloS one.

[138] Cao ZY, Mi ZM, Cheng GF, Shen WQ, Xiao X, Liu XM, Liang XT, Yu DQ (2004). Purification and characterization of a new peptide with analgesic effect from the scorpion Buthus martensi Karch. Journal of peptide.

[139] Ruan JP, Mao QH, Lu WG, Cai XT, Chen J, Li Q, Fu Q, Yan HJ, Cao JL, Cao P (2018). Inhibition of spinal MAPKs by scorpion venom peptide BmK AGAP produces a sensory-specific analgesic effect. Molecular pain.

[140] Zhao F, Wang JL, Ming HY, Zhang YN, Dun YQ, Zhang JH, Song YB (2020). Insights into the binding mode and functional components of the analgesic-antitumour peptide from Buthus martensii Karsch to human voltage-gated sodium channel 1 7 based on dynamic simulation analysis. Journal of biomolecular structure and dynamics.

[141] Song Y, Liu Z, Zhang Q, Li C, Jin W, Liu L, Zhang J, Zhang J (2017). Investigation of binding modes and functional surface of scorpion toxins ANEP to sodium channels 1 7. Toxins.

[142] Wang Y, Wang L, Cui Y, Song YB, Liu YF, Zhang R, Wu CF, Zhang JH (2011). Purification, characterization and functional expression of a new peptide with an analgesic effect from Chinese scorpion Buthus martensii Karsch (BmK AGP‐SYPU1). Biomedical chromatography.

[143] Zhang R, Yang Z, Liu YF, Cui Y, Zhang JH (2011). Purification, characterization and cDNA cloning of an analgesic peptide from the Chinese scorpion Buthus martensii Karsch (BmK AGP-SYPU2). Molekuliarnaia biologiia.

[144] Wang Y, Song YB, Yang GZ, Cui Y, Zhao YS, Liu YF, Ma Y, Wu CF, Zhang JH (2012). Arginine residues in the C-terminal and their relationship with the analgesic activity of the toxin from the Chinese scorpion Buthus martensii Karsch (BmK AGP-SYPU1). Applied biochemistry and biotechnology.

[145] Zhang R, Cui Y, Zhang X, Yang Z, Zhao Y, Song Y, Wu C, Zhang J (2010). Soluble expression, purification and the role of C-terminal glycine residues in scorpion toxin BmK AGP-SYPU2. BMB reports.

[146] Yang F, Liu S, Zhang Y, Qin C, Xu L, Li W, Cao Z, Li W, Wu Y (2018). Expression of recombinant α-toxin BmKM9 from scorpion Buthus martensii Karsch and its functional characterization on sodium channels. Peptides.

[147] Wang Y, Hao Z, Shao J, Song Y, Li C, Li C, Zhao Y, Liu Y, Wei T, Wu C, Zhang J (2011). The role of Ser54 in the antinociceptive activity of BmK9, a neurotoxin from the scorpion Buthus martensii Karsch. Toxicon.

[148] Alami M, Vacher H, Bosmans F, Devaux C, Rosso JP, Bougis PE, Tytgat J, Darbon H, Martin-Eauclaire MF (2003). Characterization of Amm VIII from Androctonus mauretanicus mauretanicus: a new scorpion toxin that discriminates between neuronal and skeletal sodium channels. Biochemical journal.

[149] Rigo FK, Bochi GV, Pereira AL, Adamante G, Ferro PR, Dal-Toé De Prá S, Milioli AM, Damiani AP, da Silveira Prestes G, Dalenogare DP, Chávez-Olórtegui C, Moraes de Andrade V, Machado-de-Ávila RA, Trevisan G (2019). TsNTxP, a non-toxic protein from Tityus serrulatus scorpion venom, induces antinociceptive effects by suppressing glutamate release in mice. European journal of pharmacology.

[150] Hoang AN, Vo HD, Vo NP, Kudryashova KS, Nekrasova OV, Feofanov AV, Kirpichnikov MP, Andreeva TV, Serebryakova MV, Tsetlin VI, Utkin YN (2014). Vietnamese heterometrus laoticus scorpion venom: evidence for analgesic and anti-inflammatory activity and isolation of new polypeptide toxin acting on Kv13 potassium channel. Toxicon.

[151] Bagheri-Ziari S, Shahbazzadeh D, Sardari S, Sabatier J-M, Pooshang Bagheri K (2021). Discovery of a New Analgesic Peptide, Leptucin, from the Iranian Scorpion, Hemiscorpius lepturus. Molecules.

[152] Golias C, Charalabopoulos A, Stagikas D, Charalabopoulos K, Batistatou A (2007). The kinin system-bradykinin: biological effects and clinical implications multiple role of the kinin system-bradykinin. Hippokratia.

[153] Rocha ESM, Beraldo WT, Rosenfeld G (1949). Bradykinin, a hypotensive and smooth muscle stimulating factor released from plasma globulin by snake venoms and by trypsin. The American journal of physiology.

[154] Ferreira S (1965). A bradykinin‐potentiating factor (BPF) present in the venom of Bothrops jararaca. British journal of pharmacology and chemotherapy.

[155] Camargo AC, Ianzer D, Guerreiro JR, Serrano SM (2012). Bradykinin-potentiating peptides: beyond captopril. Toxicon.

[156] Ferreira L, Alves E, Henriques O (1993). Peptide T, a novel bradykinin potentiator isolated from Tityus serrulatus scorpion venom. Toxicon.

[157] Meki AR, Nassar AY, Rochat H (1995). A bradykinin-potentiating peptide (peptide K12) isolated from the venom of Egyptian scorpion Buthus occitanus. Peptides.

[158] Machado RJ, Junior LG, Monteiro NK, Silva-Júnior AA, Portaro FC, Barbosa EG, Braga VA, Fernandes-Pedrosa MF (2015). Homology modeling, vasorelaxant and bradykinin-potentiating activities of a novel hypotensin found in the scorpion venom from Tityus stigmurus. Toxicon.

[159] Verano-Braga T, Figueiredo-Rezende F, Melo MN, Lautner RQ, Gomes ER, Mata-Machado LT, Murari A, Rocha-Resende C, Elena de Lima M, Guatimosim S, Santos RA, Pimenta AM (2010). Structure-function studies of Tityus serrulatus Hypotensin-I (TsHpt-I): A new agonist of B(2) kinin receptor. Toxicon.

[160] Anangi R, Koshy S, Huq R, Beeton C, Chuang WJ, King GF (2012). Recombinant expression of margatoxin and agitoxin-2 in Pichia pastoris: an efficient method for production of KV1 3 channel blockers. PloS one.

[161] Mouhat S, Visan V, Ananthakrishnan S, Wulff H, Andreotti N, Grissmer S, Darbon H, De Waard M, Sabatier JM (2005). K+ channel types targeted by synthetic OSK1, a toxin from Orthochirus scrobiculosus scorpion venom. Biochemical journal.

[162] Romi-Lebrun R, Lebrun B, Martin-Eauclaire MF, Ishiguro M, Escoubas P, Wu FQ, Hisada M, Pongs O, Nakajima T (1997). Purification, characterization, and synthesis of three novel toxins from the Chinese scorpion Buthus martensi, which act on K+ channels. Biochemistry.

[163] Han S, Yi H, Yin SJ, Chen ZY, Liu H, Cao ZJ, Wu YL, Li WX (2008). Structural basis of a potent peptide inhibitor designed for Kv1 3 channel, a therapeutic target of autoimmune disease. The Journal of biological chemistry.

[164] Tanner MR, Tajhya RB, Huq R, Gehrmann EJ, Rodarte KE, Atik MA (2017). Prolonged immunomodulation in inflammatory arthritis using the selective Kv1 3 channel blocker HsTX1[R14A] and its PEGylated analog. Clinical immunology (Orlando, Fla).

[165] Pucca MB, Bertolini TB, Cerni FA, Bordon KC, Peigneur S, Tytgat J, Bonato VL, Arantes EC (2016). Immunosuppressive evidence of Tityus serrulatus toxins Ts6 and Ts15: insights of a novel K(+) channel pattern in T cells. Immunology.

[166] Xiao M, Ding L, Yang W, Chai L, Sun Y, Yang X, Li D, Zhang H, Li W, Cao Z, Wu Y, Li J, Li S, Chen Z (2017). St20, a new venomous animal derived natural peptide with immunosuppressive and anti-inflammatory activities. Toxicon.

[167] Zhang R, Yang Z, Liu Y, Cui Y, Zhang J (2011). Purification, characterization and cDNA cloning of an analgesic peptide from the chinese scorpion Buthus martensii Karsch (BmK AGP-SYPU2). Molecular biology.

